# Novel variants of seryl-tRNA synthetase resulting in HUPRA syndrome featured in pulmonary hypertension

**DOI:** 10.3389/fcvm.2022.1058569

**Published:** 2023-01-09

**Authors:** Fan Yang, Dan Wang, Xuehua Zhang, Haoqin Fan, Yu Zheng, Zhenghui Xiao, Zhi Chen, Yunbin Xiao, Qiming Liu

**Affiliations:** ^1^Department of Cardiovascular Medicine, Second Xiangya Hospital, Central South University, Changsha, China; ^2^Department of Cardiology, Hunan Children’s Hospital, Changsha, China; ^3^Department of Ultrasound, Fujian Children’s Hospital, College of Clinical Medicine for Obstetrics & Gynecology and Pediatrics, Fujian Medical University, Fuzhou, China; ^4^Pediatrics Research Institute of Hunan Province, Hunan Children’s Hospital, Changsha, China; ^5^Department of Intensive Care Unit, Hunan Children’s Hospital, Changsha, China

**Keywords:** HUPRA syndrome, *SARS2*, missense mutation, pulmonary hypertension, genetics

## Abstract

Hyperuricemia, pulmonary hypertension, and renal failure in infancy and alkalosis syndrome (HUPRA syndrome) is an ultrarare mitochondrial disease that is characterized by hyperuricemia, pulmonary hypertension, renal failure, and alkalosis. Seryl-tRNA synthetase 2 (*SARS2*) gene variants are believed to cause HUPRA syndrome, and these variants result in the loss of function of seryl-tRNA synthetase. Eventually, mutated seryl-tRNA synthetase is unable to catalyze tRNA synthesis and leads to the inhibition of the biosynthesis of mitochondrial proteins. This causes oxidative phosphorylation (OXPHOS) system impairments. To date, five mutation sites in the *SARS2* gene have been identified. We used whole-exome sequencing and Sanger sequencing to find and validate a novel compound heterozygous variants of *SARS2* [c.1205G>A (p.Arg402His) and c.680G>A (p.Arg227Gln)], and *in silico* analysis to analyze the structural change of the variants. We found that both variants were not sufficient to cause obvious structural damage but changed the intermolecular bond of the protein, which could be the cause of HUPRA syndrome in this case. We also performed the literature review and found this patient had significant pulmonary hypertension and minor renal dysfunction compared with other reported cases. This study inspired us to recognize HUPRA syndrome and broaden our knowledge of gene variation in PH.

## 1. Introduction

Hyperuricemia, pulmonary hypertension, and renal failure in infancy and alkalosis syndrome (HUPRA syndrome) is an ultrarare mitochondrial disease that was first identified in 2011, and only eight cases have been reported worldwide. HUPRA syndrome is a mitochondrial disease featured in hyperuricemia, pulmonary hypertension, alkalosis, and renal failure (1). These four symptoms are not necessarily shown in the same patient, and the predominant clinical manifestations are different in each patient, which gives great challenge in diagnosis. As a mitochondrial disease, HUPRA syndrome cannot be cured but only has symptomatic treatments now. Research has shown that HUPRA syndrome is caused by mutations in the seryl-tRNA synthetase 2 (*SARS2*) gene, which encodes seryl-tRNA synthetase (SerRS) (2). Seryl-tRNA synthetase is responsible for the attachment of serine to the 3’-ends of tRNA*^Ser^*, which takes part in the protein synthesis and formation of respiratory chain in mitochondrial (3). The mutation of SerRS results in dysfunction of mitochondrial respiratory chain and causes mitochondrial diseases. Moreover, aminoacyl-tRNA synthetase is believed to have transcriptional effects in addition to enzyme activity. For example, recent study revealed that SerRS repressed the transcription of vascular endothelial growth factor A (VEGFA), inhibiting angiogenesis of breast cancer (4). Despite the obvious clinical relationship between SerRS and HUPRA syndrome, there is not enough genetic and experimental evidence to confirm the causality between them. Thus, it’s vital to analyze pedigree and the structure of SerRS variants to identify possible mechanism or therapies. To date, five variants of *SARS2* have been identified, however structure analysis of mutate enzyme has not been performed yet (2, 5–7). We diagnosed a HUPRA syndrome patient that carried novel compound heterozygous variants; however, in this case, pulmonary hypertension was the main clinical feature instead of renal failure. Whole-exome sequencing revealed novel compound heterozygous variants of *SARS2* (c.1205G > A and c.680G > A). Computational prediction confirmed that c.680G > A was a novel variant and was graded as variants of uncertain significance (VUS). We then performed *in silico* protein structure analysis to compare the structure of SerRS wild type and variants.

## 2. Materials and methods

### 2.1. Whole-exome sequencing and sanger sequencing

The patient’s blood was collected in EDTA anticoagulation tubes, and then DNA was extracted using a Blood Genomic DNA Mini Kit (Qiagen, Germany). Quality control of the extracted DNA was performed on a Nanodrop 2000 platform (Thermo, USA), and a Qubit 2.0 fluorometer (Thermo, USA) was used to measure the concentration of DNA. Exome capture and library establishment were performed using Agilent SureSelect Human All Exon V6 (Agilent, USA) following the manufacturer’s protocol. The library was then analyzed by an Agilent Bioanalyzer (Agilent, USA) and loaded on an Illumina HiSeq 2500 (Illumina, USA) for sequencing.

PCR was used to amplify the related exonic regions and intronic regions nearby. Sanger sequencing was then performed to validate the mutations in the proband, sister and parents.

The primers used in PCR are as follows: *SARS2*_E7-E8 (c.680G > A) F: 5′-CTGT CTCTGGAAGCTTCTATCTGG-3′, *SARS2*_E7-E8 (c.680G > A) R: 5′-TGACTAGGG CAGAGCAGAGATC-3′, *SARS2*_E14-E17 (c.1205G > A) F: 5′-CTGCCTTCTCTT CCATCGTTTCC-3′, *SARS2*_E14-E17 (c.1205G > A) R: 5′-AGTGACTGACTCT GAGGCAGCAA-3′.

### 2.2. Data filtering algorithm and large deletion/duplication analysis

Trim_galore (version 0.6.4^1^) was used to remove adapter-contaminated ends and low-quality bases with Phred scores < 20 in raw reads. Burrows-Wheeler Aligner (BWA, version 0.7.17-r1188) software (8) was used to map the polished reads with a length of ≥ 80 bp to the human reference genome (version: GRCh38), and Picard (version 2.21.1^2^) was used to remove the duplicate reads. The pipeline of the best practice of the Genome Analysis Toolkit (GATK, version 3.8) (9) was employed to refine the alignment and call variants. All variants were interpreted and classified according to the American College of Medical Genetics and Genomics (ACMG) guidelines (10) and ClinGen General Recommendations for Using ACMG/AMP Criteria by Sequence Variant Interpretation working group.^3^ The annotated variants with population frequency < 0.01 in East Asian populations in gnomAD exome or genome databases or with deleterious effects were kept and then prioritized according to the phenotype correlation score. Variants with CADD (11) Phred scores ≥ 20 or REVEL (12) scores > 0.6 were kept in high priority.

### 2.3. Region analysis and protein secondary structure analysis

The reference sequence of *SARS2* gene was NM_017827.4. The protein sequence was extracted from NCBI.^4^ It was then analyzed by T-coffee^5^ for multiple sequence alignment, Polyphen2^6^ for mutation analysis, PDB/DSSP Snapshot 25-May-2021 (included in the Polyphen2 analysis bundle) for 3D structure, SOPMA^7^ for secondary structure prediction, and Missense 3d^8^ is used for structure damage assessment.

### 2.4. *In silico* protein structure analysis

The 3D structure of SerRS was downloaded from Uniprot database,^9^ and the Uniprot ID was Q9NP81. PyMOL 2.5.2 was deployed to acquire the 3D structure of p.Arg402His, p.Arg227Gln, and p.Arg402His/p.Arg227Gln variants.

To acquire the stable conformation of the variants, we used AMBER 18 software to perform molecule dynamics simulation. First, variants were described using ff14B force field. Then, hydrogen atoms, a truncated octahedral TIP3P solvent box, Na^+^ and Cl^–^ were added to the system. Before stimulation, we used 2500-step steepest descent method and 2500-step conjugate gradient method to optimize the system energy. Then the system was heated for 200 ps under constant volume and heating rate, which allowed the system increasing from 0 to 298.15 K, and the temperature was maintained at 298.15 K. In order to further distribute the solvent molecules in the solvent box, a 500 ps NVT phylogenetic simulation was deployed. Finally, a 500 ps equilibrium simulation was deployed under isothermal and isobaric condition. During simulation process, the non-bond truncation distance was set to 10 A. The PME method was used to calculate the long-range electrostatic interaction, and SHAKE method was used to constrain hydrogen atoms. Langevin algorithm was used for temperature control: collision frequency was set to 2 ps^–1^. The pressure of the system was set to 1 atm, and integration step was set to 2 fs^–1^. The trajectories were saved every 10 ps for analysis.

## 3. Clinical manifestations

The reported case was a 9-month-old male infant from China with the clinical manifestations of fatigue and tachypnoea. The patient reached full-term delivery, and no abnormality was detected during pregnancy. The patient’s mother had an acute upper respiratory tract infection during pregnancy, but no treatments were applied. The patient exhibited hoarseness, mental slowing, snoring, and choking on milk since 4.5 months of age, and the patient suffered from recurrent pneumonia before this admission. The patient had exclusive breast feeding before 7-month-old, and complementary foods were introduced at 7-month-old.

**FIGURE 1:**
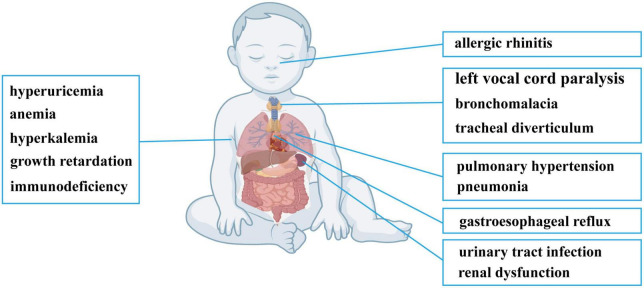
Clinical findings of the patient with HUPRA syndrome.

After admission, physical examination revealed a fever of 38°C, heart rate of 160 bpm, cold clammy limbs, moist rales on both lungs and loud P2 heart sounds. Electronic bronchoscopy found left vocal cord paralysis, laryngomalacia, tracheal diverticulum, left main bronchomalacia and endobronchitis. A CT scan showed exudative lesions on both lungs, left and right principal bronchus stenosis, and enlargement of the right ventricle, right atrium and main pulmonary artery. Echocardiography also showed enlargement of the right ventricle, right atrium and main pulmonary artery, accompanied by tricuspid regurgitation velocity of 437 cm/s, tricuspid regurgitation gradient of 76 mmHg, and estimated pulmonary artery systolic pressure (PASP) of 86 mmHg. The manifestations on echocardiography and CT are summarized in Supplementary Figure 1. Blood tests found a decreased absolute number of T lymphocytes and T helper lymphocytes and a decrease in T helper cells/T suppressor cells. Besides, neutrophil percentage was elevated, and monocyte percentage was normal. He had elevated TnT, proBNP, CK-MB, serum K^+^, serum uric acid, serum BUN and serum creatinine levels and decreased carbon dioxide combining power (CO_2_CP), serum Cl^–^ and serum Na^+^ levels (Table 1). Urinalysis revealed increases in white blood cell (WBC), red blood cell (RBC), urine protein and bacterial counts, and urine culture confirmed *Klebsiella pneumoniae* infection. Screening for inherited metabolic disease was normal. The main clinical manifestations of the patient are summarized in Figure 1. Whole-exome sequencing revealed novel compound heterozygous variants of *SARS2* (c.1205G > A and c.680G > A). There were no similar conditions in relatives of the patient, although the patient’s father suffered from hyperuricemia.

**TABLE 1:** Laboratory data of the patient with HUPRA syndrome.

Variable	Patient’s data	Normal range
Hemoglobin, g/L	96↓	97.0–141.0
Neutrophils percentage, /%	68.3↑	9.00–57.00
Monocyte percentage, /%	8.1	2.00–13.00
Lymphocyte percentage, /%	20.7↓	31.00–81.00
T helper lymphocyte percentage, /%	13.54↓	25–50
Th/Ts	0.49↓	0.70–2.80
NT-proBNP, pg/ml	1474↑	0.0–125.0
CK-MB, pg/ml	5.2↑	0.00–4.87
TnT, pg/ml	164.3↑	< 14
Serum uric acid, μmol/L	473↑	119–416
Serum Na^+^, mmol/L	133↓	134–143
Serum Cl^–^, mmol/L	96↓	98–110
CO_2_CP, mmol/L	20↓	22.00–29.00
Serum BUN, mmol/L	9.2↑	1.1–5.9
Serum creatinine, umol/L	40↑	13–33
urinary WBC, /μl	316.6↑	0–9.2
urinary bacteria counts, /μl	14254.8↑	0–94.0

Subsequently, the patient was treated with ceftriaxone for urinary tract infection and pneumonia, and the symptoms were significantly relieved. Urinalysis returned to normal, and the exudative lesions on both lungs were partially absorbed. Sildenafil, bosentan, furosemide, and oxygen inhalation were applied for pulmonary hypertension, and the estimated PASP decreased to 59 mmHg. Mitochondria support treatment was also applied; this treatment included vitamin B1, vitamin B2, vitamin B6, coenzyme Q10, levocarnitine, mecobalamin, and acetylcysteine.

At the latest follow-up, the patient gained significant relief. Hemoglobin, WBC absolute number and lymphocyte percentage were all normal. BNP, CK-MB, cTnI, serum UA and serum creatinine levels were all normal. The latest estimated PASP was 45 mmHg.

## 4. Genetic testing

**FIGURE 2:**
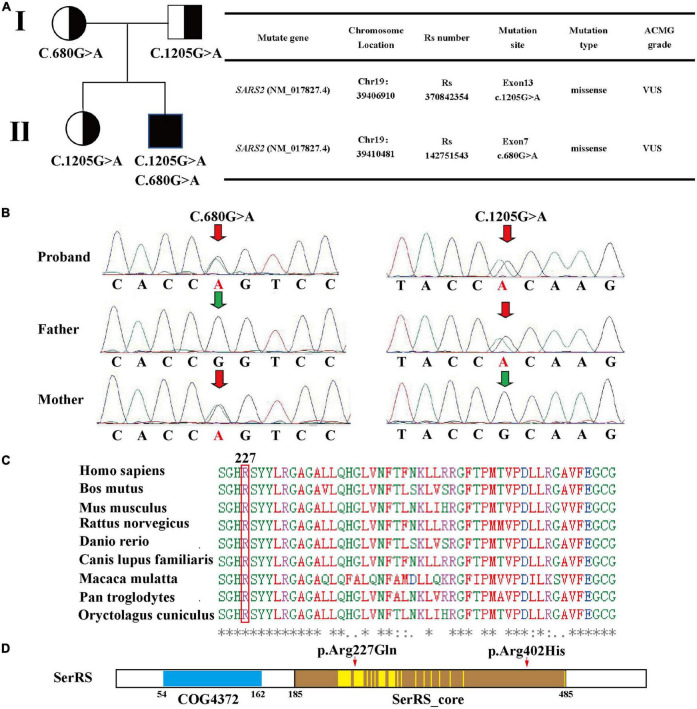
**(A)** Family pedigree, mutation sites, and ACMG grade based on whole-exome sequencing. **(B)** Sanger sequencing validation of the patient. **(C)** Multiple sequence alignment of 227-Arg using T-coffee. **(D)** Region analysis of seryl-tRNA synthetase 2 using NCBI, two conserved domain is identified: COG4372 (54–162) and SerRS_Core (185–485). According to the dual-mode recognition of SerRS, 36 of the residues are located in the dimer-interface (marked in yellow highlight), and 227-Arg is one of these residues.

Novel compound heterozygous variants of *SARS2* (c.1205G > A and c.680G > A) were identified by whole-exosome sequencing and Sanger sequencing (Figures 2A, B). The c.1205G > A (p.Arg402His) variant has been reported in previous case reports, and it was the most common *SARS2* variant in HUPRA syndrome. However, c.680G > A (p.Arg227Gln) was a novel variant. According to ACMG standards and ClinGen General Recommendations for Using ACMG/AMP Criteria by Sequence Variant Interpretation working group, we categorized c.1205G > A (p.Arg402His) as VUS (PM2, PP3, PP4) and c.680G > A (p.Arg227Gln) as VUS (PM2, PP3, PM3, PP4). To confirm the pathogenicity of c.680G > A, we performed computational prediction using the PROVEAN and PolyPhen-2 online tools. The results showed that c.680G > A had a probability of pathogenicity (Supplementary Figures 2A, B). Multiple sequence alignment was performed, and the results showed that R227 was located in the conserved region of SerRS (Figure 2C).

Then, we performed Sanger sequencing of the proband, parents and sister (Figure 2B and Supplementary Figure 2C). This revealed that the patient’s father and sister harbored the c.1205G > A variant, and the mother harbored the c.680G > A variant. However, these three individuals did not present typical symptoms of HUPRA syndrome, and their serum uric acid, renal function, blood tests, echocardiography were all normal (Table 2). Thus, we believe that a single heterozygous mutation of *SARS2* is not sufficient to cause HUPRA syndrome.

**TABLE 2:** Laboratory data of the family.

Variable	Mother’s data	Father’s data	Normal range
Hemoglobin, g/L	139	149	110.0–150.0 (female) 120.0–160.0 (male)
Lymphocyte percentage, /%	49.6↑	37.2	20.00–40.00
Neutrophils percentage, /%	40.4↓	56.1	50.00–70.00
Monocyte percentage, /%	4.1	3.9	3.00–12.00
NT-proBNP, pg/ml	16.50	17.60	0.0–125.0
TnI, ng/ml	0.031	0.032	< 0.16
Serum uric acid, umol/L	259.16	361.08↑	142–340
Serum Na^+^, mmol/L	146.20	143.80	135–150
Serum Cl^–^, mmol/L	102.80	101.90	97–110
Serum BUN, mmol/L	3.47	3.42	2.6–7.5
Serum creatinine, μmol/L	58.44	68.73	41–114
Echocardiography	Normal	Normal	–

## 5. Literature review

We then performed literature review to summarize *SARS2* variants and clinical manifestations of HUPRA syndrome. To date, eight HUPRA syndrome patients and five kinds of variants of *SARS2* have been reported. Variants and clinical manifestations are summarized in Table 3 (2, 5–7). All patients exhibit progressive renal failure except this case, and this case is the only compound heterozygous variants patient that prominently presents pulmonary hypertension phenotype. The literature review indicated that compound heterozygous variants tended to show milder symptoms than homozygous variants, in which patient 6 carried compound heterozygous variants had the longest life span and this case got significantly relieved after treatment. We also noticed that c.1205G > A + c.1205G > A variants exhibit progressive renal failure and pulmonary hypertension, c.1205G > A + c.667G > A variants showed progressive renal failure without pulmonary hypertension, and c.1205G > A + c.680G > A showed pulmonary hypertension without progressive renal failure.

**TABLE 3:** Comparison of clinical manifestation of HUPRA cases.

Patient	1	2	3	4	5	6	7	8 (this case)
Age, (month-old)	4	7	4	15	2	52	ND	9
Sex	Male	Female	Female	Female	Male	Female	Female	Male
Premature delivery	+, 34 weeks	+, 27 weeks	+, 27 weeks	−, 37 weeks	+, 36 weeks	+, 36 weeks	ND	−, 39 weeks
Anemia, (Hb, g/dl)	9 (*N* > 10)	4.8 (*N* > 10)	ND	8.4 (*N* > 12)	7.6 (*N* > 12)	8.7 (*N* > 11)	+	9.6 (*N* > 9.7)
Hyperuricemia, (mg/dl)	13.8 (*N*, 2.4–6.4)	26.8 (*N*, 2.4–6.4)	14.1 (*N*, 2.4–6.4)	11 (*N*, 2.2–7)	9.4 (*N*, 2.2–7)	11 (*N*, 2.4–6.6)	+	7.94 (*N*, 2.0–7.0)
PASP (mmHg)	60	67	Increased	ND	Increased	–	Increased	86
Hypertension	+	+	ND	+	+	–	ND	–
Renal failure	+	+	+	+	+	+	+	Mild
Serum creatinine, (mg/dl)	0.99 (*N*, 0.2–0.4)	1.14 (*N*, 0.2–0.4)	ND	1.01 (*N*, 0.35–0.5)	0.96 (*N*, 0.35–0.5)	1.24 (*N*, 0.17–1.02)	Increased	0.45 (*N*, 0.16–0.41)
Serum BUN, (mg/dl)	44 (*N*, 5–18)	87 (*N*, 5–18)	ND	159 (*N*, 5–18)	ND	129 (*N*, 15–40)	ND	25.7 (3.1–16.5)
NT-proBNP, (pg/ml)	ND	ND	ND	ND	ND	ND	ND	1474 (*N* < 125.0)
Hyponatremia, (mEq/l)	116 (*N*, 133–146)	124 (*N*, 133–146)	122 (*N*, 133–146)	ND	+	ND	ND	133 (*N*, 134–143)
Hypomagnesemia, (mg/dl)	0.9 (*N*, 1.58–2.4)	1.2 (*N*, 1.58–2.4)	0.97 (*N*, 1.58–2.4)	ND	1.7 (*N*, 1.5–2.3)	1.29 (*N*, 1.29–2.70)	ND	2.02 (*N*, 1.94–2.92)
Immunodeficiency	Leukopenia	Leukopenia	ND	–	–	–	Significant	+
Maldevelopment	+	+	+	+	+	+	+	+
Echocardiography	Left ventricular hypertrophy	Biventricular hypertrophy	Right ventricular hypertrophy	ND	Right ventricular hypertrophy	ND	ND	Right ventricular hypertrophy
Metabolic alkalosis	+	+	+	+	+	–	+	–
Cause of death	Multiorgan failure	Respiratory insufficiency	Pulmonary hypertension	Multiorgan failure	Cardiac failure	Renal failure	–	–
Mutation sites	c.1169A > G homozygous	c.1169A > G homozygous	c.1169A > G homozygous	c.1205G > A homozygous	c.1205G > A homozygous	c.1205G > A c.667G > A	c.515A > G homozygous	c.1205G > A c.680G > A
PMID	21255763	21255763	21255763	24034276	24034276	33751860	35445976	This case

## 6. Secondary structure analysis

We find that variant p.Arg227Gln (this case) is close to site p.Val223Met (patient 6), and both patients show relatively mild symptoms. One reason for this is that the region containing Arg-227 and Val-223 is not in the synthetase core domain (3, 7) (Figure 2D), and another reason for this is that the precise treatment delays the progression of the disease.

However, this case showed significant pulmonary hypertension that responded well to sildenafil and bosentan, while patient 6 suffered from renal failure without pulmonary hypertension. To find the possible reasons for the difference, we conducted secondary structure prediction of SerRS, which revealed that p.Arg227Gln caused different secondary structure changes from those caused by p.Val223Met (Supplementary Figure 3A). The results shows that p.Arg227Gln causes secondary structure changes other than a single amino acid residue substitution. These results also partially explain the differences of these variants. However, it’s still unclear whether these changes cause severe damage to SerRS structure. Thus, we used missense 3D online tools to evaluate the effect of the variants detected in patient 8. However, the results show that both p.Arg402His and p.Arg227Gln variants cannot cause structural damage to SerRS (Supplementary Figure 2D).

## 7. *In silico* analysis of SerRS variants

**FIGURE 3:**
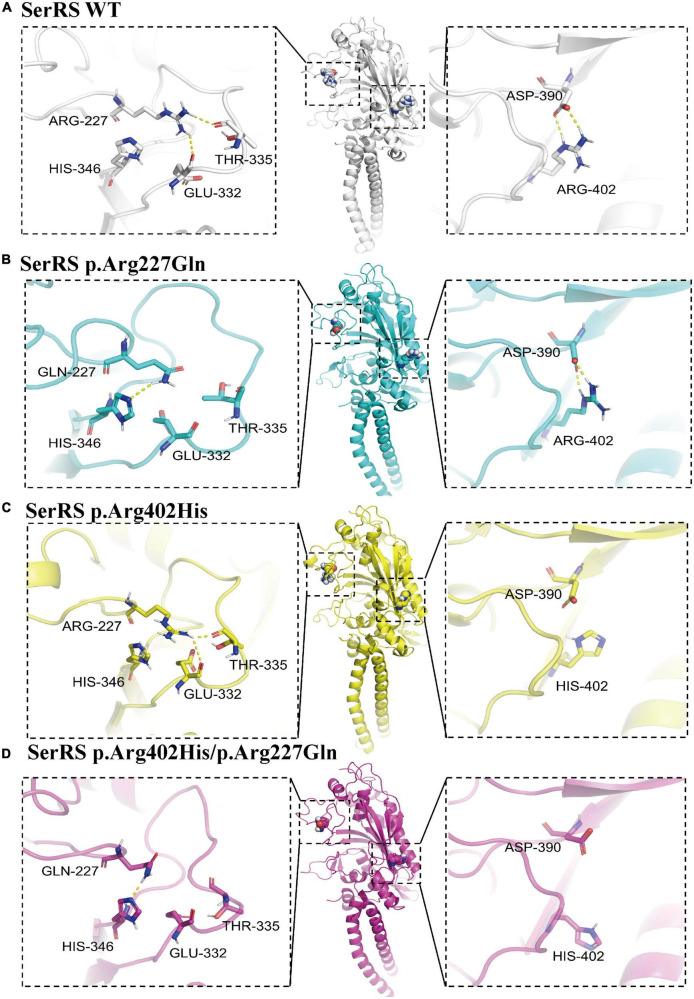
The structure analysis of SerRS variants using PyMOL 2.5.2, and the structure is optimized by molecule dynamics simulation to acquire the most stable configurations. The hydrogen bonds are marked with yellow dashes. **(A)** Configuration of SerRS wild type. **(B)** Configuration of SerRS p.Arg227Gln variant. **(C)** Configuration of SerRS p.Arg402His variant. **(D)** Configuration of SerRS p.Arg402His/p.Arg227Gln variants. All variants did not cause significant structure damage to SerRS. However, p.Arg227Gln and p.Arg402His/p.Arg227Gln variants lost the hydrogen bond of Arg-227 with Thr-335 and Glu-332, while acquired the hydrogen bond with His-346. p.Arg402His and p.Arg402His/p.Arg227Gln lost the hydrogen bond and ionic bonds between Arg-402 and Asp-390.

Although p.Arg402His and p.Arg227Gln variants could not cause structural damage to SerRS. We hypothesized that the substitution of amino acid might affect intermolecular forces such as hydrogen bond, which changed conformation of the enzyme or caused instability of the structure. Thus, we used PyMOL 2.5.2 and AMBER 18 software to acquire p.Arg402His, p.Arg227Gln, and p.Arg402His/p.Arg227Gln variants conformations and run molecule dynamics simulation. Results confirmed that all variants did not cause significant structure damage to SerRS. However, p.Arg227Gln and p.Arg402His/p.Arg227Gln variants lost the hydrogen bond of Arg-227 with Thr-335 and Glu-332, while acquired the hydrogen bond with His-346 (Figure 3), which might cause the instability of the loop. Meanwhile, p.Arg402His and p.Arg402His/p.Arg227Gln lost the hydrogen bond and ionic bonds between Arg-402 and Asp-390 (Figure 3). In summary, although these variants could not cause severe structure damage, they changed the intermolecular bond which might affect the stability of SerRS.

## 8. Discussion

HUPRA syndrome is an ultrarare mitochondrial disease, *SARS2* gene mutations are believed to cause HUPRA syndrome. *SARS2* gene mutations result in SerRS loss of function that leads to an inhibition of the biosynthesis of mitochondrial proteins, then results in an impaired oxidative phosphorylation (OXPHOS) system. To date, including this case, there are 5 kinds of variants of *SARS2* (2, 5–7). Based on former reports, homozygous variants tend to exhibit all symptoms of HUPRA syndrome. c.1205G > A + c.667G > A variants showed hyperuricemia and severe renal failure, and c.1205G > A + c.680G > A variants showed significant pulmonary hypertension and mild renal dysfunction, and c.680G > A was a novel variant of *SARS2*. We found that in all cases, a single heterozygous variant did not result in HUPRA syndrome, and these carriers were healthy or had hyperuricemia. This proves that HUPRA syndrome is an autosomal recessive genetic disease. We also noticed that *SARS2* gene splicing defect caused progressive spastic paresis instead of HUPRA syndrome (13). We found that all variants causing HUPRA syndrome were single amino acid substitution, and our results showed p.Arg402His/p.Arg227Gln variants could not cause structure damage, but the variants causing progressive spastic paresis had completely changed C-terminal. Although splicing efficiency between neuronal and other organs might partially explain difference (13), the exact mechanism was unknown.

The predominant organ affected by HUPRA syndrome and other mitochondrial diseases is usually the kidney, especially renal tubules, which require a large amount of ATP for reabsorption functions. However, *SARS2* gene splicing defect causes progressive spastic paresis instead of HUPRA syndrome., but we found that all variants causing HUPRA syndrome were single amino acid substitution. Variants in our case could not result in structure damage, but only changed the intermolecular bond, but the variants causing progressive spastic paresis had completely changed C-terminal. Although splicing efficiency between neuronal and other organs might partially explain difference, the exact mechanism was unknown. In this case, pulmonary hypertension was the principal symptom instead of renal failure, and the patient was older than most former cases. It is possible that different variants of *SARS2* cause different domain changes of SerRS, and in this case, we inferred that the remaining enzyme activity was sufficient to maintain renal functions thus far. HUPRA syndrome is a mitochondrial disease that presents as pulmonary hypertension (1), which indicates a connection between *SARS2* and pulmonary vascular remodeling. One possible reason for this is that the impaired OXPHOS system stimulated glycolysis, which resulted in the proliferation and migration of pulmonary smooth muscle cells (PASMCs) (14–16). Another possible reason for this is that SerRS not only participates in the mitochondrial respiratory chain but also regulates pathways related to pulmonary hypertension. A study conducted in HEK293 cells and HUVECs found that seryl-tRNA synthetase could bind to the promoter region of VEGFA, which inhibited c-myc and HIF-1α binding to the promoter and deceased the transcription of VEGFA (17). Mutated or phosphorylated SerRS lost its binding ability to the VEGFA promoter and resulted in VEGFA upregulation. VEGFA is one of the most important factors that stimulate pulmonary artery endothelial cells and PASMC proliferation (18). Due to lack of research, whether mutations of *SARS2* result in pulmonary hypertension *via* VEGFA pathways remains unknown and deserves further research. Furthermore, hypoxia induces the phosphorylation of SerRS and promotes upregulation of VEGFA and remodeling of the pulmonary artery, which suggests that it is vital to correct hypoxia in controlling the progression of pulmonary hypertension in HUPRA syndrome. Thus, we combined oxygen inhalation with sildenafil and bosentan in the treatments of this case, which successfully decreased PASP.

Hyperuricemia is believed to originate from renal failure in mitochondrial diseases (7). However, in this case, there was no evidence of renal failure, except for hyperuricemia, and at present, the cardiovascular symptoms were quite severe in this case. Research found that uric acid was elevated in pulmonary hypertension and ventricle hypertrophy, which has been used as biomarker of prognosis in PH (19, 20). Another possible explanation was that *SAR*S2 gene mutations may affected enzyme activity of purine metabolism.

Despite the typical symptoms of HUPRA syndrome, we also found that the patient had decreased lymphocyte counts, elevated neutrophils counts and normal monocyte counts. The abnormality of lymphocytes returned to normal after treatments. It’s unclear whether the prophylaxis for respiratory tract infection was related to the abnormality of the immune system. We reviewed all reported HUPRA cases and found that half of these cases had significant immunodeficiency, which presented mainly as leukopenia. Currently, there is no research on the relationship between *SARS2* and the immune system. However, previous studies indicated that mitochondrial dysfunction resulted in maturation arrest of immune cells in bone marrow, which caused decreased immune cells in peripheral blood and immunodeficiency (21). However, it’s unclear how or to what extent *SARS2* variants affect immune system.

Currently, there are no targeted therapies for HUPRA syndrome and most mitochondrial diseases (5, 22), and most of these patients die of multiorgan failure in the early stage. The main therapies are supportive and symptomatic. According to the mechanism of HUPRA syndrome, we raised a series of managements to improve the survival and quality of life of the patient, including PH targeted therapy, oxygen inhalation, B vitamins, coenzyme Q10, γ-globulin and L-carnitine, which require follow-up for evaluation and adjustments. This study inspired us to recognize HUPRA syndrome, notice its different phenotypes and broaden knowledge of gene variation in PH.

## 9. Conclusion

We reported a novel *SARS2* compound heterozygous variants c.1205G > A/c.680G > A. Genetic analysis revealed that c.680G > A was in conserved region and caused changes in SerRS protein structure. In this patient, p.Arg402His and p.Arg227Gln were both graded as VUS and this patient had significant pulmonary hypertension and minor renal dysfunction compared with other reported cases. However, the detailed mechanism needs further research.

## Data availability statement

The data presented in this study are deposited in the NCBI Sequence Read Archive (SRA), BioProject accession number: PRJNA915330, SRA accession number: SRP414628.

## Ethics statement

Written informed consent was obtained from the individual(s), and minor(s)’ legal guardian/next of kin, for the publication of any potentially identifiable images or data included in this article.

## Author contributions

All authors listed have made a substantial, direct, and intellectual contribution to the work, and approved it for publication.

## Footnotes

1
http://www.bioinformatics.babraham.ac.uk/projects/trim_galore/

2
https://broadinstitute.github.io/picard/

3
https://www.clinicalgenome.org/working-groups/sequence-variant-interpretation/

4
https://www.ncbi.nlm.nih.gov/nuccore/NM_017827.4/

5
https://www.ebi.ac.uk/Tools/msa/tcoffee/

6
http://genetics.bwh.harvard.edu/pph2/

7
https://npsa-prabi.ibcp.fr/cgi-bin/npsa_automat.pl?page=npsa_sopma.html

8
http://missense3d.bc.ic.ac.uk/missense3d/

9
https://www.uniprot.org/uniprotkb/Q9NP81/entry#names_and_taxonomy
