# Supplementary figures and images for: Novel variants of seryl-tRNA synthetase resulting in HUPRA syndrome featured in pulmonary hypertension
Source: Front Cardiovasc Med. 2023 Jan 9;9:1058569. doi: 10.3389/fcvm.2022.1058569 (PMC9868236; doi:10.3389/fcvm.2022.1058569)

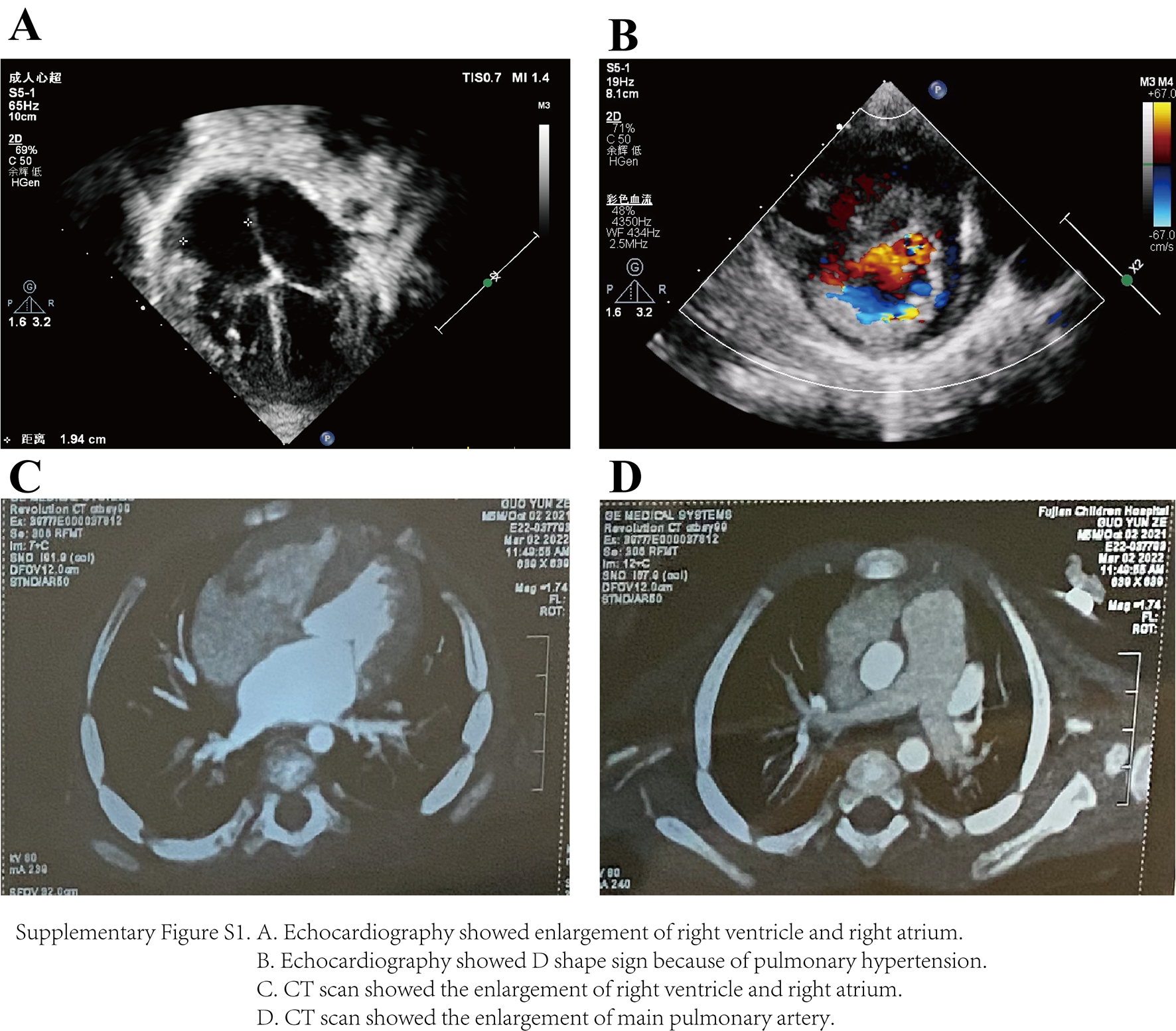

Supplement: Supplementary file 1 [file Image_1.TIF]

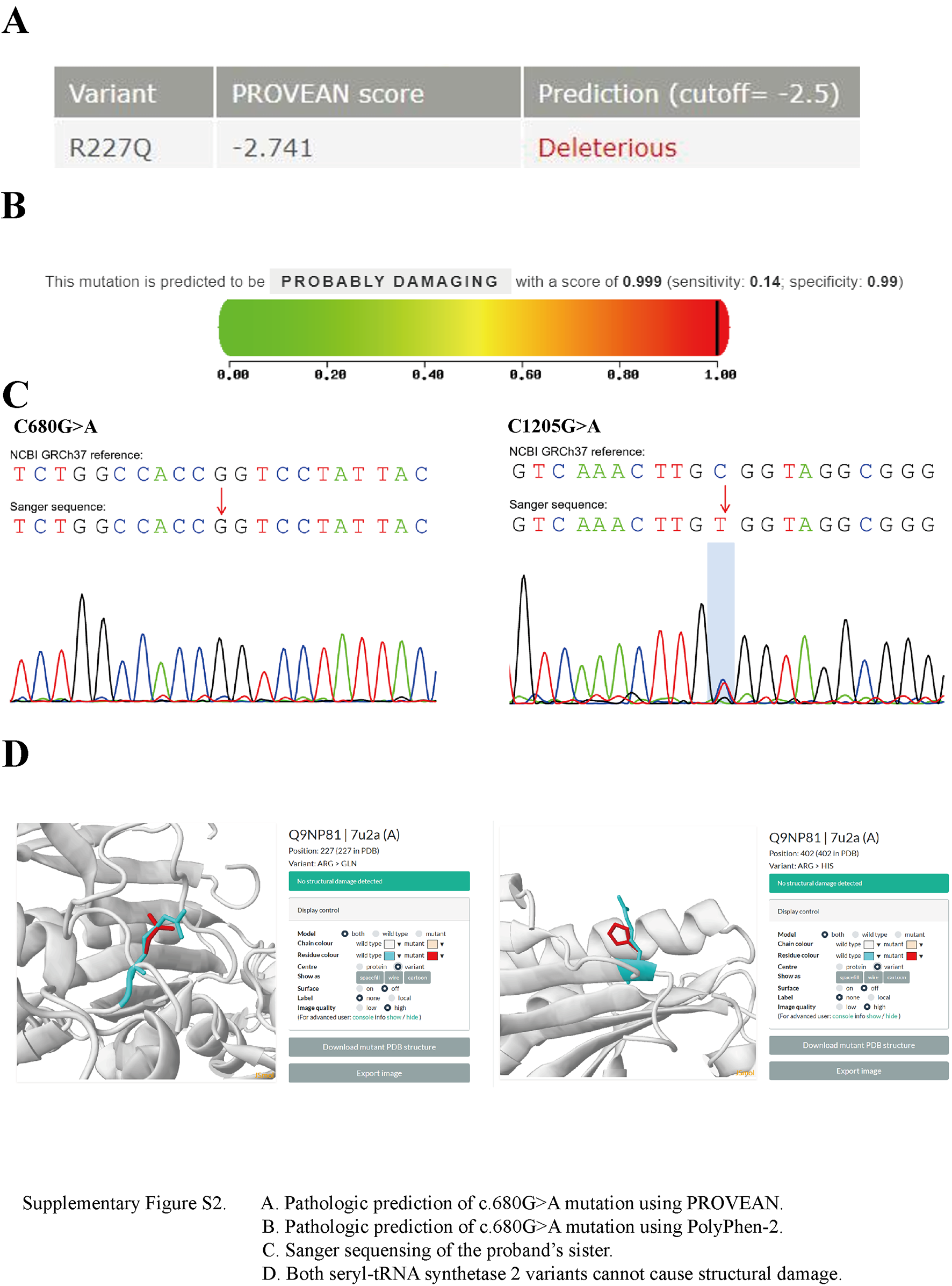

Supplement: Supplementary file 2 [file Image_2.TIF]

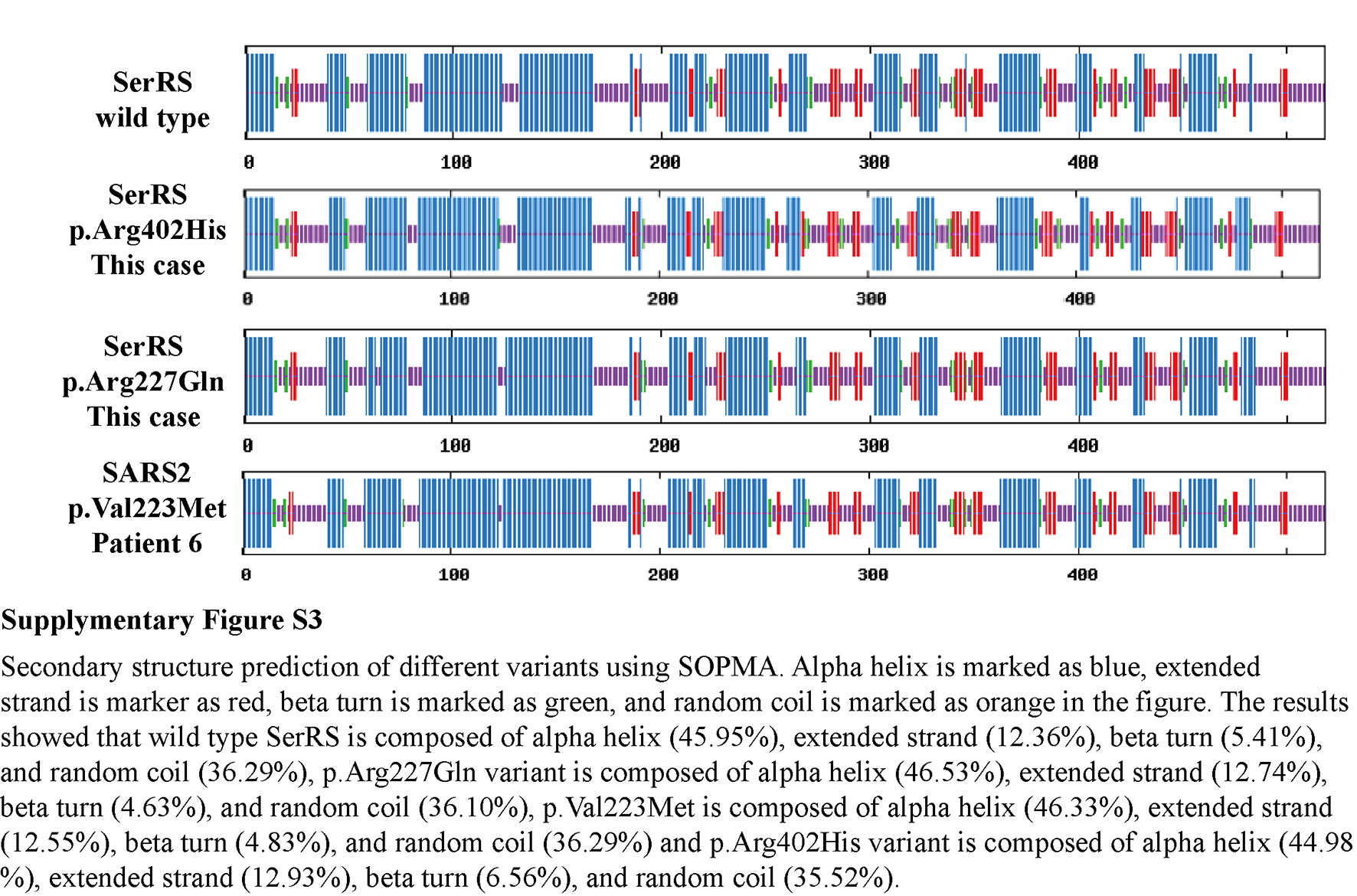

Supplement: Supplementary file 3 [file Image_3.tif]
